# Spatial distribution of settlement of *Diadema antillarum* around Saba, Dutch Caribbean

**DOI:** 10.7717/peerj.17291

**Published:** 2024-04-30

**Authors:** Oliver J. Klokman, Alwin Hylkema

**Affiliations:** 1 University of Applied Sciences Van Hall Larenstein, Leeuwarden, The Netherlands; 2 Marine Animal Ecology group, Wageningen University and Research, Wageningen, The Netherlands

**Keywords:** Echinoid, Sea urchin, Windward, Leeward, Hydrodynamics

## Abstract

The mass mortality event of the herbivorous sea urchin *Diadema antillarum* in 1983–1984 has been a major contributor to the diminished resilience of coral reefs throughout the Caribbean. The reduction in grazing pressure resulted in algae proliferation, which inhibited coral recruitment after disturbances such as disease, hurricanes, pollution and climatic change induced marine heat waves. Natural recovery of *D. antillarum* after the 1983–1984 die-off has been slow. However, the few locations with recovered populations exhibit signs of improvement in coral reef health, prompting interest in *D. antillarum* restoration. Current restoration strategies include translocation of wild individuals, the restocking of juveniles that are either cultured from gametes or collected as settlers and head-started in a nursery, and assisted natural recovery by providing suitable settlement substrate. Both the collection of wild settlers and assisted natural recovery necessitate an understanding of the local, spatiotemporal trends in settlement. In this study, which was carried out on the Dutch Caribbean Island of Saba, artificial turf settlement collectors were deployed at nine locations around the island and monitored from June 2019 till July 2020 (13 months). The primary objective was to identify trends in larval settlement in space and time, to be able to optimize restoration efforts. Additionally, the small size of Saba allowed us to deploy settlement collectors around the island and compare *D. antillarum* settlement between windward and leeward sides. Our study showed that on Saba, *D. antillarum* settlement peaked in June and July, following similar seasonal trends observed around other islands in the Northeastern Caribbean. By far the most settlement occurred at the leeward side of the island, suggesting that hydrodynamic forces entrained *D. antillarum* larvae in the lee of Saba and/or calmer waters facilitated settlement. Limited settlement occurred on the more exposed windward locations. The identified high settlement locations are candidates for settler collection and restoration attempts. Continued monitoring of *D. antillarum* settlement, especially in light of the 2022 *D. antillarum* die-off, holds significance as it can provide insights into the potential of natural recovery.

## Introduction

The long-spined black sea urchin, *Diadema antillarum* (Philippe, 1845), was once a ubiquitous herbivore on Caribbean reefs (Lessios, 2016) and exerted significant force in shaping the development of reef flora and fauna (Carpenter, 1981; Sammarco, 1982; Bak, Carpay & De Ruyter Van Steveninck, 1984). In 1983 and 1984, *D. antillarum* suffered a mass-mortality caused by an unidentified pathogen that affected its entire geographic range (Bak, Carpay & De Ruyter Van Steveninck, 1984; Lessios, Robertson & Cubit, 1984; Lessios, 1988a; Hughes, 1994). This significantly diminished the grazing pressure that *D. antillarum* exerted, resulting in immediate and enduring shifts in community structure (Hughes et al., 2010; Lessios, 2016). The biomass of turf and macroalgae increased substantially, particularly in the shallow areas where *D. antillarum* used to be most abundant (Lessios, 1988a), at the costs of crustose coralline algae (CCA) and coral (Hughes, 1994; Edmunds & Carpenter, 2001).

Over the subsequent four decades, Caribbean coral reefs continued to change, not just because of the lack of grazing but also due to diseases, hurricanes, pollution and climate change (Jackson et al., 2014; De Bakker et al., 2017; Cramer et al., 2021). Areas once characterized by scleractinian corals, CCA and shortly cropped algal turfs, became dominated by macroalgae, cyanobacteria and other coral competitors. The absence of grazing pressure has resulted in poor coral recruitment, reducing the resilience of Caribbean coral reefs (Hughes et al., 2010). Recovery of *D. antillarum* following the 1983–1984 die-off remained slow and sporadic (Lessios, 2016). In 2022, a more recent die-off further reduced *D. antillarum* numbers throughout the Eastern Caribbean and Greater Antilles (Hylkema et al., 2023; Levitan, Best & Edmunds, 2023), although the effects of this die-off appeared to have been less severe compared to the 1983–1984 mortality, as populations in parts of the Caribbean remained unaffected.

Before the 2022 die-off, a few populations of *D. antillarum* had recovered to such extent that the increased grazing pressure reversed the earlier observed phase-shift (Edmunds & Carpenter, 2001; Debrot & Nagelkerken, 2006; Miller et al., 2007; Rogers & Lorenzen, 2016). These sites observed reduced algal cover (Myhre & Acevedo-Gutiérrez, 2007; Maciá, Robinson & Nalevanko, 2007) and increased coral recruitment (Carpenter & Edmunds, 2006; Idjadi, Haring & Precht, 2010) and survival (Idjadi, Haring & Precht, 2010). Given these positive effects on the reef, various approaches to *D. antillarum* restoration have been implemented, including the translocation of wild individuals (Nedimyer & Moe, 2003; Chiappone, Swanson & Miller, 2006; Maciá, Robinson & Nalevanko, 2007; Burdick, 2008; Pilnick et al., 2023), restocking juveniles that were either cultured from gametes (Pilnick et al., 2021; Wijers et al., 2023) or collected as settlers and head-started in a nursery (Williams, 2022), and assisted natural recovery, in which barriers to natural recovery were addressed (Hylkema et al., 2022a). However, donor populations that are dense enough to sustain the removal of high numbers for translocation are rare and cultivation from gametes requires a high investment of time and expertise. Restocking with wild caught settlers and assisted natural recovery may currently be the most viable methods to actively restore *D. antillarum* in the majority of the Caribbean region. The effectiveness of these two approaches depends on the identification of locations and months with high settlement (Williams, 2016; Hylkema et al., 2022b). It is therefore essential to understand how *D. antillarum* settlement varies in time and space.

Settlement is influenced by a hierarchy of processes affecting: (1) the larval pool, (2) dispersal and (3) local (micro-)hydrodynamics, substrate availability, settlement cues, and behavior of larvae (Pineda, 2000). While regional-scale processes affecting the larval pool and transport stages have a relative high impact on settlement rates, small-scale processes such as substrate and larval behavior are known to have less impact (Rodriguez, Ojeda & Inestrosa, 1993; Pineda, 2000; Metaxas, 2013). The larval pool is mostly determined by successful spawning events. Spawning patterns in echinoderms are influenced by multiple factors, including the age and size of individuals, resource availability, lunar cycle, sea temperature, and photoperiod (Pearse & Cameron, 1991). It is clear that *D. antillarum* is an asynchronous broadcast spawner (Randall, Schroeder & Starck, 1964; Levitan, 1988) that exhibits variation in reproductive periodicity between populations (Garrido, Haroun & Lessios, 2000). Studies of spawning patterns have yielded conflicting results with some populations displaying strong synchronization with annual and lunar cycles (Lewis, 1966; Bauer, 1976; Iliffe & Pearse, 1982), while less conspicuous spawning patterns were observed in other populations (Randall, Schroeder & Starck, 1964; Lessios, 1981; Levitan, 1988). These differences in spawning patterns may be due seasonal differences between latitudes as locations with known peaks appeared to coincide with the spring (Randall, Schroeder & Starck, 1964; Lewis, 1966; Iliffe & Pearse, 1982; Williams, García-Sais & Capella, 2009) and fall (Bauer, 1976).

If fertilization is successful, larvae can disperse over long distances, potentially seeding islands several hundreds of kilometers downstream (Lessios, 1988a, 1988b; Karlson & Levitan, 1990). The presence of source populations in combination with ocean currents determine larval dispersal (Feehan et al., 2019), while food availability and predation pressure determine survival, growth, and development into competent larvae (Metaxas, 2013; Doll et al., 2022). Finally, several biological and environmental factors need to come together to allow larvae to go through metamorphosis and successfully settle (Lessios, 2016). Prior *D. antillarum* settlement studies have indicated that some areas such as Southern Florida are possibly larval limited (Miller et al., 2009; Feehan et al., 2016, 2019). Studies in other areas such as Puerto Rico (Williams, García-Sais & Yoshioka, 2011), St. Eustatius, Saba (Hylkema et al., 2022b), and Curacao (Vermeij et al., 2010; Rogers & Lorenzen, 2016) have shown that these islands have locations with comparable settlement rates to those measured around Curacao before 1983 (Bak, 1985). However, on all three islands, there were also nearby locations with much lower settlement rates (Williams, García-Sais & Yoshioka, 2011; Rogers & Lorenzen, 2016; Hylkema et al., 2022b).

These low settlement locations on islands that did not appear to be limited by the larval supply can be a result of local hydrodynamics. For example, the orientation of locations to primary winds and currents can have significant effects on marine larval accumulation with high larval retention being observed in the lee of headlands (Roughan et al., 2005; Mace & Morgan, 2006; Morgan, Fisher & Largier, 2011). On a smaller scale, Hunte & Younglao (1988), Keesing, Cartwright & Hall (1993) and Williams, García-Sais & Yoshioka (2011) observed significantly higher echinoderm settlement or recruitment on fore reef compared to back reef locations. Understanding the effects of local hydrodynamics on *D. antillarum* settlement is essential in identifying high settlement locations necessary for restoration purposes. Most *D. antillarum* studies utilized a limited number (Bak, 1985; Miller et al., 2009; Williams, Yoshioka & Garcia Sais, 2010; Maldonado-Sánchez et al., 2019) of often only leeward oriented locations (Vermeij et al., 2010; Rogers & Lorenzen, 2016; Williams, Yoshioka & Garcia Sais, 2010; Williams, García-Sais & Yoshioka, 2011; Hylkema et al., 2022b) or monitored only known temporal settlement peaks (Vermeij et al., 2010; Rogers & Lorenzen, 2016).

**Figure 1:**
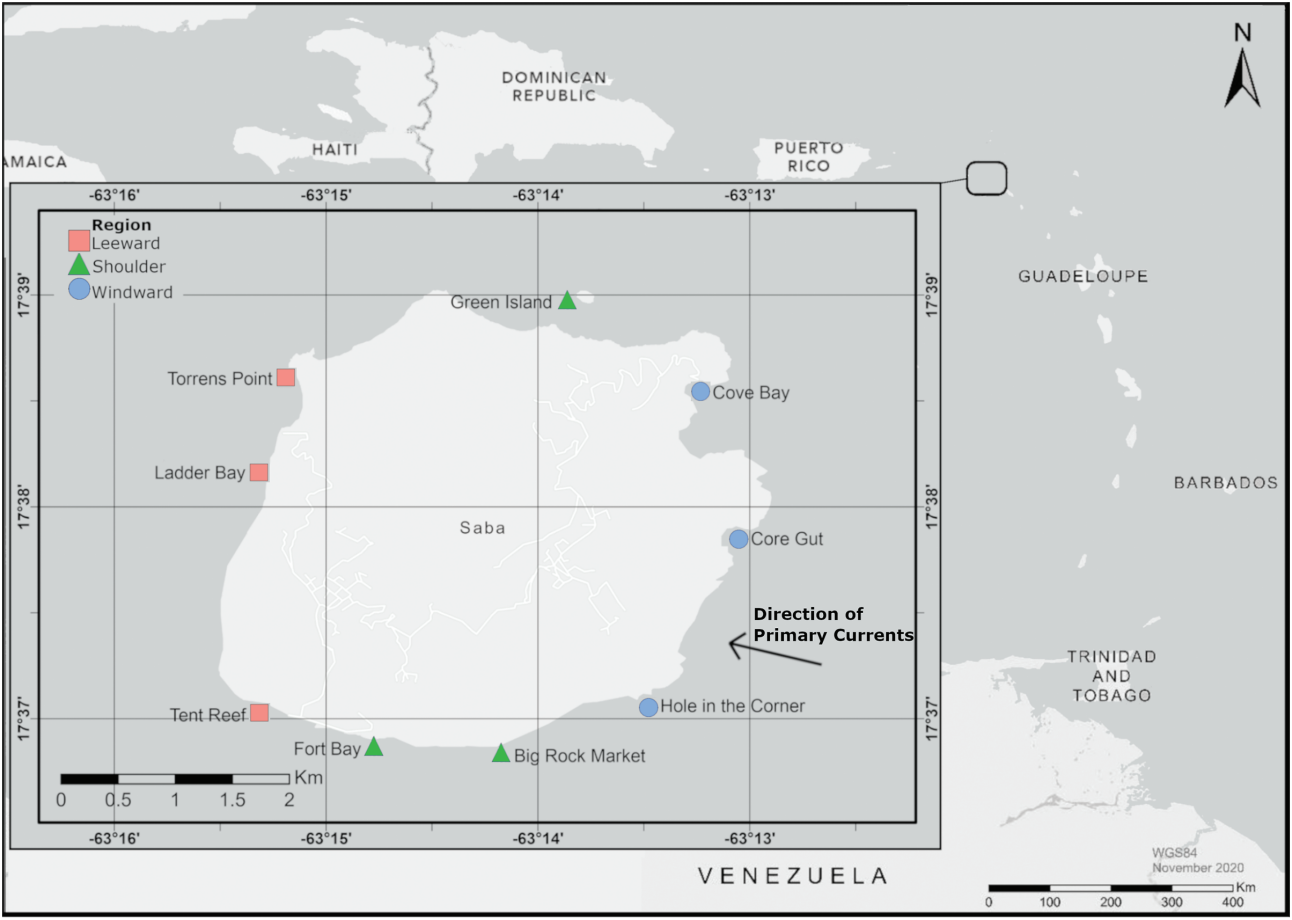
Map of experimental locations around Saba. The primary current approaches Saba from the south east, indicated by the arrow. Map created with ArcMap 10.8 using data from Esri, HERE, and Garmin. Service Layer credits: World Light Gray Base: ESRI. World Light Gray Base: ESRI, HERE, Garmin.

Saba, a Dutch Caribbean Island (Fig. 1), provided the opportunity to study island scale settlement dynamics of *D. antillarum*. Settlement is known to occur (Hylkema et al., 2022b) and, due to the small size of the island, it is logistically feasible to deploy and retrieve settlement collectors around the entire coastline. Additionally, Saba’s volcanic origin resulted in the surrounding bathymetry to be characterized by a narrow shelf surrounded by deep water (Buchan, 1998), which eliminated fore and back reef effects (Hunte & Younglao, 1988; Keesing, Cartwright & Hall, 1993; Williams, García-Sais & Yoshioka, 2011). Our study is, to our knowledge, the first to collect *D. antillarum* settlement rates around an entire island for a full year and describes spatial and temporal trends in settlement.

## Methods

*D. antillarum* settlement rates were studied monthly for a total of 13 months (from July 2019 until July 2020) at nine locations around Saba. These locations (Fig. 1) were equally spread over the western leeward side (locations Tent Reef, Ladder Bay and Torrens Point), the eastern windward side (locations Cove Bay, Core Gut and Hole in the Corner) and the northern and southern intermediate shoulder sides (location Green Island in the north and locations Big Rock Market and Fort Bay in the south) of the island. Locations were selected based on the following criteria: proximity to an existing dive site, even distribution around the island, depth ranging from 11–15 m depth and a sandy bottom for at least 5 m around the experimental location. At each location an experimental set-up modelled after Williams, García-Sais & Yoshioka (2011) and Hylkema et al. (2022b) was deployed, consisting of a line kept vertical in the water column by an anchor on the bottom and a subsurface buoy at 5 m depth. Attachment points for settlement collectors were made by making a loop in the line on 8, 8.5, 9, 9.5, and 10 m depth, as *D. antillarum* settlement is known to be highest within this depth range (Williams, García-Sais & Yoshioka, 2011). Permission to place settlement collectors for this experiment was given by Kai Wulf from the Saba Conservation Foundation, the Saba National Marine Park (SNMP) manager at the time of this study.

**Figure 2:**
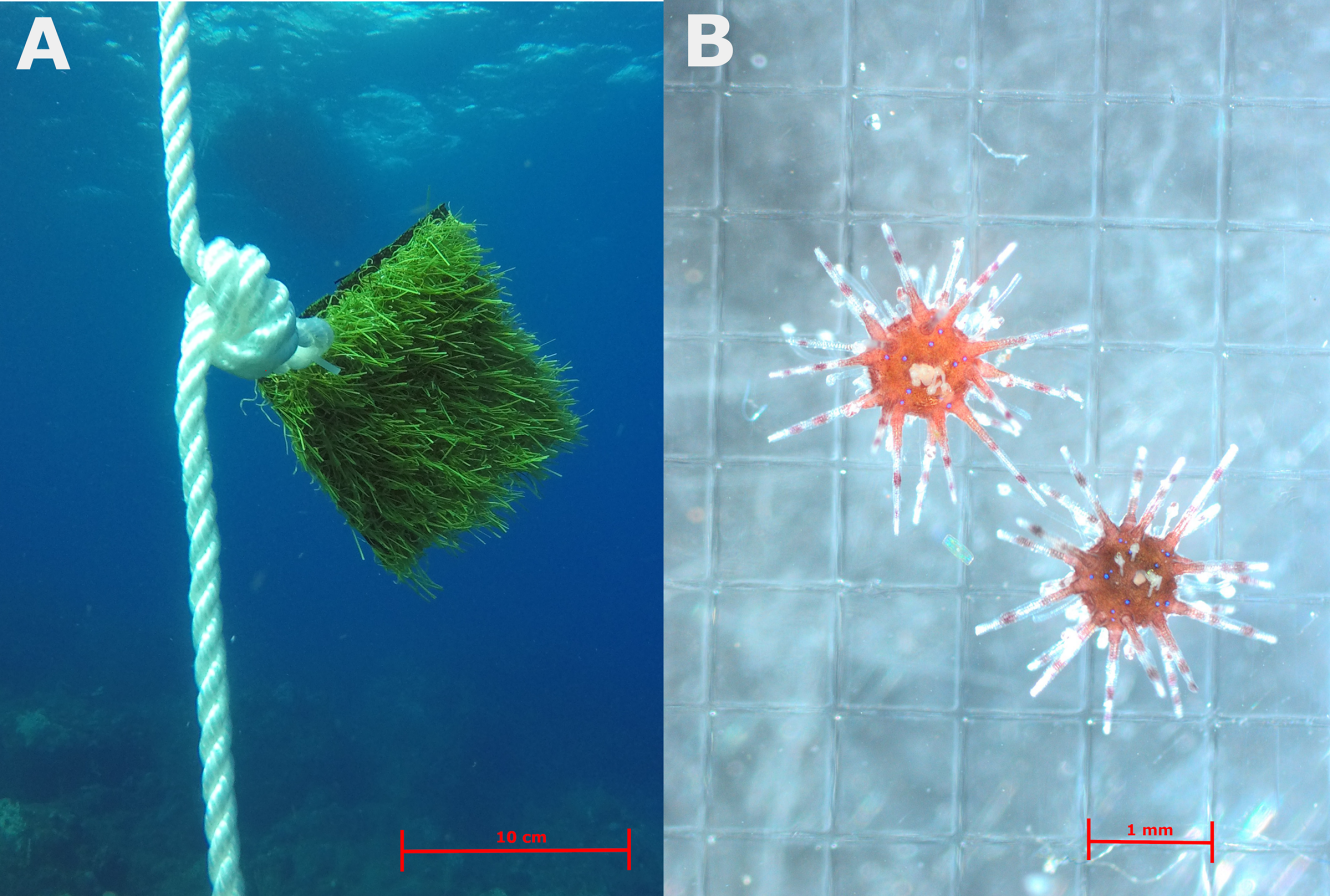
(A) Example of artificial turf settlement collectors suspended in the water column. (B) Recently settled *Diadema antillarum* collected from the collectors.

Settlement collectors consisted of artificial turf, a material proven to be suitable for *D. antillarum* settlement (Hylkema et al., 2022b). Two 10 × 10 cm pieces of polyethylene and polypropylene artificial turf with a blade height of 1.5 cm were tie wrapped back-to-back to create a settlement collector (Fig. 2A). Collectors were tie-wrapped to the loops in the rope and retrieved monthly using SCUBA. Each collector was carefully enclosed underwater with a Ziploc bag and immediately replaced with a new collector. Analysis of each collector took place within 2 h of collection by thoroughly rinsing the sample five times in different white trays, after which all trays and the collector were inspected counting all *D. antillarum* settlers (Hylkema et al., 2022a). The white trays allowed relatively easy identification of *D. antillarum* as their propensity to attach to the tray prevented oscillation with sediments and other organisms and provided high contrast against their bright red color, as seen in Fig. 2B.

### Data analyses

Statistical analyses were performed with R (R Core Team, 2022) using Rstudio version 1.4.1717 (RStudio Team, 2023). To account for slight differences in soaking time between months, the settlement rate was expressed as monthly settlement rate following the methodology of Williams, Yoshioka & Garcia Sais (2010) and Hylkema et al. (2022b). The *D. antillarum* counts on each collector were divided by the number of days the collector was submerged and multiplied by 30 (average of 1 month).

GLMs with a Poisson distribution (“glm” function of the “MASS” package (Venables & Ripley, 2002)) were used to model the monthly settlement rate (response variable). Two different models were constructed: a regional model considering the explanatory variables region (leeward, windward or shoulder), depth, and month and a location model in which region was replaced with location. Based on the Akaike Information Criterion (AIC), which was used to select the models with the best fit (Zuur et al., 2009), depth was excluded from both models. There were not enough degrees of freedom to include interactions in the models. Model validation revealed no overdispersion, and plotted residuals showed no obvious patterns. Tukey’s *post-hoc* tests, utilizing estimated marginal means from the R package “emmeans” (Lenth, 2023), were conducted to examine differences between locations or regions as well as months. Graphs were generated using the R package “ggplot2” (Wickham, 2016). *P* values <0.05 were considered statistically significant.

## Results

From July 2019 through July 2020, we collected 509 *D. antillarum* settlers from all locations around Saba. Pooled counts per region resulted in 354 settlers at leeward locations, 111 at shoulder locations, and 44 at the windward locations. Mean settlement rates were peaking at the start of monitoring in July 2019, ranging between 0 and 20 settlers per collector, depending on location. Mean monthly settlement rates decreased during the subsequent summer months of August and September, approached zero by November and remained zero throughout the winter months of December through March. The first settlers of 2020 appeared in April and varied per month and per location in the concurrent months. Average number of *D. antillarum* per collector, per location and per month can be found in Table S1.

**Figure 3:**
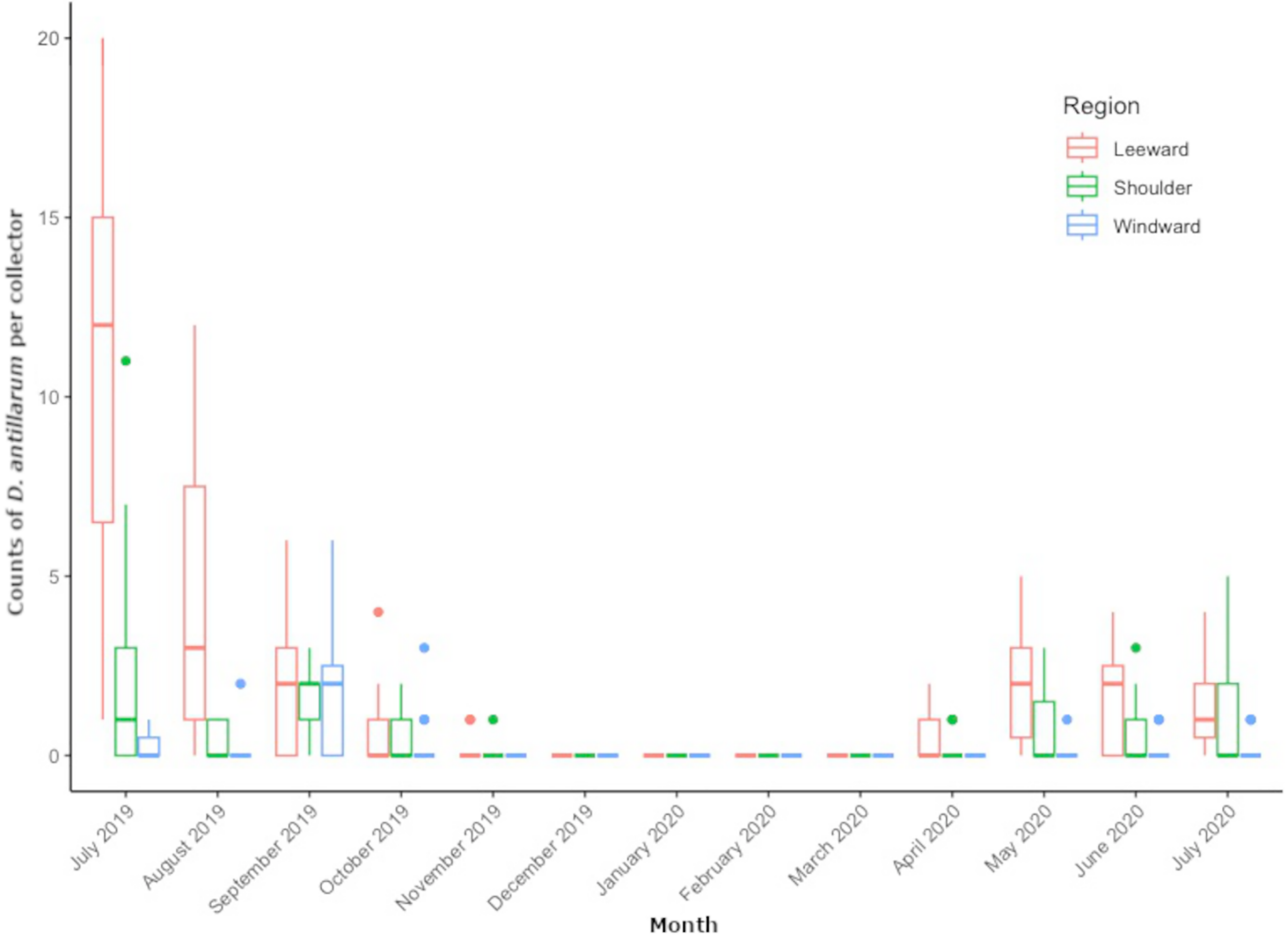
Monthly Diadema antillarum settler counts per collector of locations pooled by region. The boxplot shows median (bold line), the first and third quartiles (colored box outline), and the lower and upper extremes, colored dots represent outlying values (>1.5 interquartile range from third quartile).

Figure 3 shows the distribution of monthly regional settlement counts. Both month (LRT = 363.76, df = 12, *P* < 0.001) and region (LRT= 849.42, df = 2, *P* < 0.001) were significant predictors of settlement. Pairwise comparisons revealed significantly higher settlement in the leeward region (*P* = < 0.001) compared to the shoulder and windward region, while the shoulder region had significantly higher settlement compared to the windward region (*P* < 0.001). Pairwise comparisons between months revealed settlement rate to decrease in subsequent months from July 2019 to November 2019 (*P* < 0.001 for each month) until settlement rate reached zero. Settlement rates per region did not significantly increase again until May 2020 (*P* = 0.008). Monthly settlement rate did not significantly differ between May 2020 and July 2020.

In the location specific analysis, month (LRT = 851.93, df = 12, *P* < 0.001) and location (LRT = 424.39, df = 8, *P* < 0.001) were significant predictors of *D. antillarum* settlement. Pairwise comparisons between locations revealed significantly higher settlement at leeward locations Torrens Point and Ladder Bay (*P* < 0.001) and significantly lower settlement at windward locations Core Gut and Cove Bay (*P* < 0.001) when compared to all other locations. Significantly more settlement was recorded at the Green Island (*P* = 0.0047 for both comparisons) and Tent Reef (*P* < 0.001 for both comparisons) compared to the locations Hole in the Corner and Big Rock Market. Pairwise comparisons of settlement rate between months at specific locations were similar to the results of the regional analysis and showed a significant decrease in subsequent months from July 2019 to November 2019 (*P* < 0.001 for each month) when settlement approached zero. Settlement rates per location did not significantly increase again until May 2020 (*P* = 0.008). Between May, June and July 2020 there were no significant differences in settlement rate. Comparisons between July 2019 and July 2020 revealed significantly higher settlement rate in July 2019 (*P* < 0.001).

## Discussion

The seasonal *D. antillarum* settlement observed in the present study, with high settlement in the summer period from July 2019 to September 2019 and low settlement in winter period from December 2019 to March 2020, is similar to observations around St. Eustatius in 2019 (Hylkema et al., 2022b) and Puerto Rico in 2006 (Williams, Yoshioka & Garcia Sais, 2010). The maximum monthly settlement rate per m^2^ (calculated by dividing the settlement rate per collector by the planar surface of the collector, 0.02 m^2^) found in the present study was 1,153 *D. antillarum* per m^2^, comparable to settlement rates recorded on mid-water collectors in 2006 and 2008 in Puerto Rico and 2019 on St. Eustatius where mean maximum rates depending on location were 1,100, 220, and 760 *D. antillarum* per m^2^, respectively (Williams, Yoshioka & Garcia Sais, 2010; Williams, García-Sais & Yoshioka, 2011; Hylkema et al., 2022b). Variation in annual reproductive periodicity between conspecific populations of *D. antillarum* has been revealed in multiple studies (Garrido, Haroun & Lessios, 2000). Similar settlement peaks between adjacent islands or islands at similar latitudes likely reflect the similarities in spawning. Seasonal differences in primary phytoplankton production within Caribbean waters influenced by riverine nutrient inputs (Müller-Karger et al., 1989; Forget et al., 2011; López et al., 2013) and/or seasonal temperature changes may act as spawning stimuli. Phytoplankton blooms peaking between June and August (López et al., 2013) could supply the necessary nutrients for larvae to reach competency and successfully complete metamorphosis, potentially accounting for increased settlement rates during the April to October period. Settlement in July 2020 was lower compared to the same month in 2019. Inter-annual variation within echinoid populations is common, with settlement numbers varying by several orders of magnitude between years being recorded for other echinoid species (Balch & Scheibling, 2000). Therefore, differences in settlement between July 2019 and July 2020 may be attributed to natural fluctuations.

Region and location were significant determinants of *D. antillarum* settlement. Settlement rates were highest at the western leeward locations, while windward locations exhibited the lowest settlement rates. Intermediate settlement rates were observed at the shoulder regions of the island. Successful echinoderm settlement and recruitment is complex and dependent on a series of biotic and abiotic factors aligning at each life stage (Lessios, 2016), with spatiotemporal patterns of settlement being linked to larval supply (Metaxas, 2013). While successful spawning at upstream locations and dispersal driven by oceanographic currents might have the biggest effect on the larval supply (Pineda, 2000; Doll et al., 2022), island-scale differences as observed in the present study likely resulted from local oceanographic features (Rodriguez, Ojeda & Inestrosa, 1993). Echinopluteus larvae are considered weak swimmers with control over dispersal and settlement at only relatively small spatial scales (Metaxas, 2013; Doll et al., 2022) consequently behaving more like passive particles (Wing et al., 1995). Under laboratory conditions, *D. antillarum* larvae have shown a very limited range of motion and were dependent on water movement to maintain position in the water column (Pilnick et al., 2021; Wijers et al., 2023), indicating the importance of hydrodynamic forces in determining settlement. Features such as eddies, gyres, and current systems can either promote larval dispersal or retain larvae (Carrillo et al., 2017).

Saba is an island characterized by rough waters and strong currents with the prevailing North Equatorial Current and trade winds arriving from the southeast (Buchan, 1998). The primary currents wrap around Saba flowing in a westward direction before joining again on the western side of the island, indicated in Fig. 1. This flow around the island likely results in downstream effects including the formation of eddies, which can result in the mechanical entrainment and retention of marine plankton (Hamner & Hauri, 1981; Wolanski, Imberger & Heron, 1984). High local entrainment of larvae in the eddies of reef systems has been described by many authors for a variety of organisms including larval fish (Hunte & Younglao, 1988; Rodríguez et al., 2001) coral larvae (Harriott & Fisk, 1988; Boehlert, Watson & Sun, 1992; Cowen & Castro, 1994), zooplankton (Alldredge & Hamner, 1980; Le Borgne, Dandonneau & Lemasson, 1985; Hernández-León, 1991; Hernández-León et al., 2001, Landeira et al., 2010), echinoids (Scheibling & Mladenov, 1987), and phytoplankton (Heywood & Priddle, 1987).

**Figure 4:**
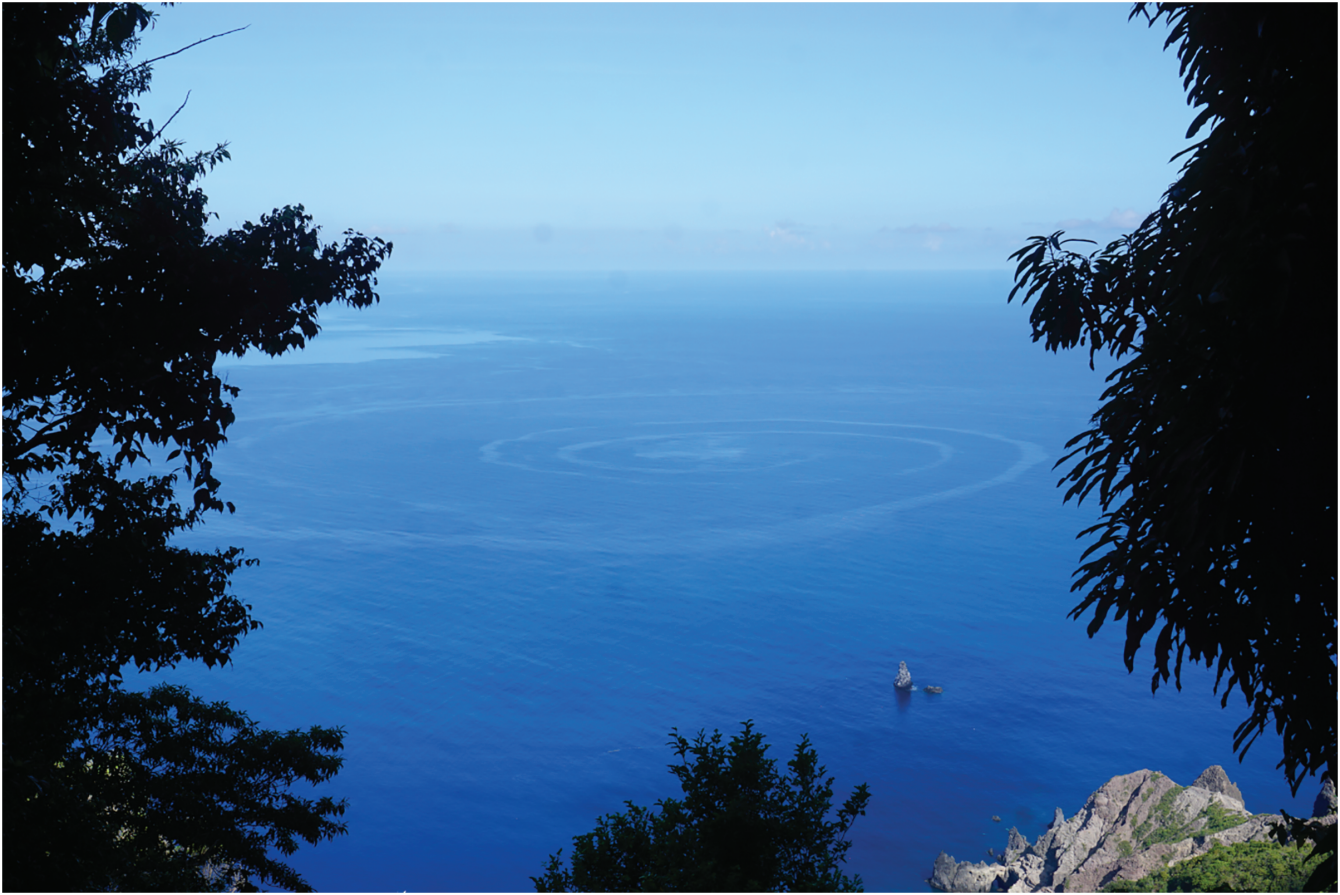
Temporary sub meso-scale eddy observed at the Torrens Point location on the west side of Saba.

Stationary eddies can entrain and aggregate larvae, facilitating high local recruitment (Criales & Lee, 1995). Eddy entrainment does not necessarily hinder larval development as upwelling and downwelling dynamics can provide adequate food availability (Lane et al., 2003). Non-stationary eddies are well established as an important mechanism of larval transport and have been linked to influxes of *D. antillarum* in the Florida Keys (Feehan et al., 2019). The lee of Saba includes the locations Ladder Bay and Torrens Point which experienced the highest settlement in this study. An eddy system here would explain the higher observed settlement, as the chance of settlement increases if larvae are retained and accumulated in the same area. During periods of calm weather, sub mesoscale eddies have been observed in this area, such as off the coast of Torrens Point (indicated in Fig. 4), supporting this hypothesis. Furthermore, the Green Island location which is situated in the lee of an islet, may undergo similar eddy induced larvae retention. Since eddies are not fixed features, often dissipating and moving over time (Feehan et al., 2019), they may also contribute to the inter-annual variability of settlement observed on Saba.

An alternative explanation for low settlement at some windward locations is the influx of *Sargassum* biomass. Regional increases in *Sargassum* spp. biomass have led to the formation of large floating mats that accumulate on beaches and in bays and lagoons throughout the Caribbean and the Gulf of Mexico (Gower, Young & King, 2013). The proliferation of *Sargassum* is likely attributed to the combination of high oceanic temperatures and nutrient inputs coupled with changes in surface current (Cabanillas-Terán et al., 2019). As the mats accumulate, they decompose and remove dissolved oxygen and decrease water quality to the extent that fish die-offs are reported. The effects of the mats are three-fold: they create an inhospitable environment for sensitive *D. antillarum* larvae when decomposing (Pilnick et al., 2021), can affect rates of settlement on other substrates as *Sargassum* is a known settlement inducing cue (Wijers et al., 2024), and, finally, *D*. antillarum settlement in *Sargassum* mats likely results in high mortality as the mats harbor an abundance of potential predators (Coston-Clements et al., 1991). At the windward locations Cove Bay and Core Gut, sargassum mats have been observed to periodically accumulate (Personal observations, both authors) and the potential effect of these accumulations on settlement rates cannot be disregarded.

The rough waters around Saba are an additional potential determinant of *D. antillarum* settlement. Considering the poor swimming ability of *D. antillarum* larvae, locations with high turbulence and flow can prevent larvae from interacting with physical and chemical cues needed to induce settlement (Rodriguez, Ojeda & Inestrosa, 1993). While the larvae of some urchin species can have settlement spurred when encountering high turbulence (Gaylord, Hodin & Ferner, 2013), other species’ settlement appeared to be linked to periods of upwelling relaxation (Wing et al., 1995). Settlement responses of larvae to differing hydrodynamic traits can have species specific effects likely tied to adult life histories (Hodin, Ferner & Gaylord, 2020). This is likely the case for *D. antillarum*, which has had settlement linked to lulls in wave height and shear stress (Maldonado-Sánchez et al., 2019). The apparent necessity of calm water conditions for settlement may explain why recovered *D. antillarum* populations are often reported in shallow and sheltered conditions at leeward locations, which is for example the case in Curacao (Debrot & Nagelkerken, 2006; Rogers & Lorenzen, 2016), Dominican Republic (Torres, 2015) and Puerto Rico (Rodríguez-Barreras et al., 2014). Therefore, portions of coastline where primary winds and currents relax can result in enhanced settlement. On the windward eastern side (Cove Bay, Core Gut, Hole in the Corner) and the shoulder locations (Big Rock Market, Fort Bay and Green Island), there is near continuous high wind and wave exposure. In contrast, the leeward western locations (Tent Reef, Ladder Bay, Torrens Point) are more sheltered, possibly resulting in increased settlement rates.

Predation is considered the most significant cause of echinoderm larval mortality (Metaxas, 2013). There is strong evidence that during the planktonic larval stages of many species, predation is variable, nonrandom, and frequently high (Vaughn & Allen, 2010). Thus, the abundance and distribution of *D. antillarum* larvae around Saba may be influenced by variation in planktonic predation pressure between locations. However, the relatively high settlement rates on downstream locations suggests upstream locations were not larvae limited and other factors were more important in determining settlement. Apart from larval mortality, predation is also known to be a significant source of post-settlement mortality of benthic invertebrates (Hunt & Scheibling, 1997), including *D. antillarum* (Lessios, 1988b; Harborne et al., 2009). Post settlement predation on the settlement collectors, especially the role of co-settling micro-predators such as crabs is not well understood (Williams, García-Sais & Yoshioka, 2011). Predation studies are necessary to test the effects of predation on *D. antillarum* settlement rates on collectors, however, for ophiuroid settlers it has been demonstrated that suspended collectors mitigated potential post settlement predation Balch & Scheibling (2000) and Wing et al. (1995) found no evidence that crab predation controlled red urchin settlers on settlement collectors. This makes it less likely that post-settlement predation was a primary cause of the significant spatial trends observed in the present study.

Our study is the first to include *D. antillarum* settlement rates all around an island, including both leeward and windward locations. The observed pattern in settlement rates, in which high settlement peaks were observed within the lee of the island and little settlement was observed at the windward locations of the island can be the result of a complex interplay between predation, location specific conditions, and hydrodynamic features. However, due to the similar conditions in which the settlement collectors were deployed, it is likely that hydrodynamic conditions determined both the number of larvae available for settlement and the actual ability of larvae to settle. A study investigating *D. antillarum* larval abundances in the waters around Saba could corroborate this hypothesis. The identified settlement hotspots in the lee of Saba are valuable for restoration purposes, as these locations are suitable for collecting settlers for head-starting and restocking and may also be suitable for interventions addressing barriers in natural recovery. Our study also serves as a baseline, as mass mortalities of *D. antillarum* were observed on many islands in the Eastern Caribbean and Greater Antilles in 2022 (Hylkema et al., 2023; Levitan, Best & Edmunds, 2023) and the decline in adult *D. antillarum* populations may once again curtail larvae availability and settlement rates in the region.

## Supplemental Information

10.7717/peerj.17291/supp-1 Supplemental Information 1 Raw settlement data. The data collected and location characteristics. The dates of deployment and retrieval of settlement collectors and the counts of diadema settlers at retrieval. These counts were used in statistical analysis to compare months and sites for settlement.

10.7717/peerj.17291/supp-2 Supplemental Information 2 Average number of *D. antillarum* settlers collected per collector per location and per month. ± denotes standard error

10.7717/peerj.17291/supp-3 Supplemental Information 3 Model validation values for location and region analysis.

10.7717/peerj.17291/supp-4 Supplemental Information 4 R script of data analysis.

## Additional Information and Declarations

### Field Study Permissions

The following information was supplied relating to field study approvals (*i.e*., approving body and any reference numbers): Permission for this study was given verbally by the Saba Conservation Foundation (SCF) Manager Kai Wulf.

### Data Availability

The following information was supplied regarding data availability: The raw data are available in the Supplemental Files.
