# Supplementary material for: Spatial distribution of settlement of Diadema antillarum around Saba, Dutch Caribbean
Source: PeerJ. 2024 Apr 30;12:e17291. doi: 10.7717/peerj.17291 (PMC11067890; doi:10.7717/peerj.17291)
Supplement: Supplemental Information 2 — ± denotes standard error [file peerj-12-17291-s002.docx]

Supplementary Table 1. Average number of D. antillarum settlers per collector per month, per location, and in total expressed with ± standard error.

|  | Fort Bay | Tent Reef | Ladder Bay | Torrens Point | Green Island | Cove Bay | Core Gut | Hole in the Corner | Big Rock Market | **Total** | |
| --- | --- | --- | --- | --- | --- | --- | --- | --- | --- | --- | --- |
| July 2019 | 0.6 ± 0.6 | 6.4 ± 2.4 | 14.2 ± 2.0 | 16.6 ± 2.7 | 6.2 ± 1.8 | 0 ± 0 | 0.2 ± 0.2 | 0.6 ± 0.2 | 0.6 ± 0.4 | 45.4 ± 1.0 | |
| August | 0.6 ± 0.2 | 0.8 ± 0.4 | 6.8 ± 1.8 | 5.8 ± 1.9 | 0.2 ± 0.2 | 0 ± 0 | 0 ± 0 | 0.4 ± 0.4 | 0.4 ± 0.2 | 15.0 ± 0.5 | |
| September | 0.6 ± 0.2 | 0.4 ± 0.2 | 2.6 ± 0.5 | 0.8 ± 0.4 | 1.0 ± 0.0 | 0.4 ± 0.2 | 0.8 ± 0.4 | 2.0 ± 0.5 | 1.2 ± 0.2 | 9.8 ± 0.1 | |
| October | 0.2 ± 0.2 | 1.4 ± 0.9 | 1.0 ± 0.3 | 0 ± 0 | 0.8 ± 0.5 | 0.2 ± 0.2 | 0.6 ± 0.6 | 0.2 ± 0.2 | 0.8 ± 0.6 | 5.2 ± 0.2 | |
| November | 0.2 ± 0.2 | 0.2 ± 0.2 | 0 ± 0 | 0.2 ± 0.2 | 0 ± 0 | 0 ± 0 | 0 ± 0 | 0 ± 0 | 0 ± 0 | 0.6 ± 0.0 | |
| December | 0 ± 0 | 0 ± 0 | 0 ± 0 | 0 ± 0 | 0 ± 0 | 0 ± 0 | 0 ± 0 | 0 ± 0 | 0 ± 0 | 0 ± 0 | |
| January 2020 | 0 ± 0 | 0 ± 0 | 0 ± 0 | 0 ± 0 | 0 ± 0 | 0 ± 0 | 0 ± 0 | 0 ± 0 | 0 ± 0 | 0 ± 0 | |
| February | 0 ± 0 | 0 ± 0 | 0 ± 0 | 0 ± 0 | 0 ± 0 | 0 ± 0 | 0 ± 0 | 0 ± 0 | 0 ± 0 | 0 ± 0 | |
| March | 0 ± 0 | 0 ± 0 | 0 ± 0 | 0 ± 0 | 0 ± 0 | 0 ± 0 | 0 ± 0 | 0 ± 0 | 0 ± 0 | 0 ± 0 | |
| April | 0.6 ± 0.2 | 0.2 ± 0.2 | 1.4 ± 0.4 | 0.2 ± 0.2 | 0 ± 0 | 0 ± 0 | 0 ± 0 | 0 ± 0 | 0 ± 0 | 2.4 ± 0.1 | |
| May | 0.6 ± 0.6 | 2.2 ± 0.7 | 2.6 ± 0.7 | 1.2 ± 0.6 | 0.8 ± 0.5 | 0.2 ± 0.2 | 0 ± 0 | 0 ± 0 | 0.8 ± 0.6 | 8.4 ± 0.2 | |
| June | 1.6 ± 0.5 | 1.6 ± 0.4 | 0.6 ± 0.6 | 2.6 ± 0.5 | 0 ± 0 | 0 ± 0 | 0 ± 0 | 0.6 ± 0.2 | 0.2 ± 0.2 | 7.2 ± 0.2 | |
| July 2020 | 1.2 ± 0.5 | 1.6 ± 0.7 | 1.0 ± 0.5 | 1.2 ± 0.4 | 1.8 ± 0.7 | 0 ± 0 | 0.4 ± 0.2 | 0.2 ± 0.2 | 0 ± 0 | 7.4 ± 0.2 | |
| **Total** | 6.2 ± 0.1 | 14.8 ± 0.3 | 30.2 ± 0.5 | 28.6 ± 0.6 | 10.8 ± 0.3 | 0.8 ± 0.0 | 2.0 ± 0.1 | 4.0 ± 0.1 | 4.0 ± 0.1 | |  |
