# Supplementary material for: Spatial distribution of settlement of Diadema antillarum around Saba, Dutch Caribbean
Source: PeerJ. 2024 Apr 30;12:e17291. doi: 10.7717/peerj.17291 (PMC11067890; doi:10.7717/peerj.17291)
Supplement: Supplemental Information 3 [file peerj-12-17291-s003.docx]

Supplementary Table 2A

Location analysis model validation

glm(`rounded monthly settlement rate`~ Loc + Month + Depth)

single term deletions tested:

| Variable | Df | Deviance | AIC | LRT | Pr(>Chi) |
| --- | --- | --- | --- | --- | --- |
| null |  | 540.85 | 1019.5 |  |  |
| Loc | 8 | 965.24 | 1427.9 | 424.39 | <2e-16 |
| Month | 12 | 1390.14 | 1844.8 | 848.29 | <2e-16 |
| Depth | 4 | 549.97 | 1020.6 | 9.11 | 0.05839 |

Final model:

glm(`rounded monthly settlement rate`~ Loc + Month)

Supplementary Table 2B

Region analysis model validation

glm(`rounded monthly settlement rate`~ Region + Month + Depth)

single term deletions tested:

| Variable | Df | Deviance | AIC | LRT | Pr(>Chi) |
| --- | --- | --- | --- | --- | --- |
| null |  | 601.49 | 1068.1 |  |  |
| Region | 2 | 965.24 | 1427.9 | 363.76 | <2e-16 |
| Month | 12 | 1450.74 | 1893.4 | 849.25 | <2e-16 |
| Depth | 4 | 610.60 | 1069.2 | 9.11 | 0.05839 |

Final model:

glm(`rounded monthly settlement rate`~ Region + Month)
